# Leveraging Janus Substrates as a Confined “Interfacial Reactor” to Synthesize Ultrapermeable Polyamide Nanofilms

**DOI:** 10.34133/research.0359

**Published:** 2024-04-29

**Authors:** Cheng-Ye Zhu, Hao-Nan Li, Bian-Bian Guo, Yu Fang, Chang Liu, Hao-Cheng Yang, Chao Zhang, Hong-Qing Liang, Zhi-Kang Xu

**Affiliations:** ^1^ MOE Engineering Research Center of Membrane and Water Treatment Technology, and Key Lab of Adsorption and Separation Materials & Technologies of Zhejiang Province, Department of Polymer Science and Engineering, Zhejiang University, Hangzhou 310058, China.; ^2^ The “Belt and Road” Sino-Portugal Joint Lab on Advanced Materials, International Research Center for X Polymers, Zhejiang University, Hangzhou 310058, China.

## Abstract

Porous substrates act as open “interfacial reactors” during the synthesis of polyamide composite membranes via interfacial polymerization. However, achieving a thin and dense polyamide nanofilm with high permeance and selectivity is challenging when using a conventional substrate with uniform wettability. To overcome this limitation, we propose the use of Janus porous substrates as confined interfacial reactors to decouple the local monomer concentration from the total monomer amount during interfacial polymerization. By manipulating the location of the hydrophilic/hydrophobic interface in a Janus porous substrate, we can precisely control the monomer solution confined within the hydrophilic layer without compromising its concentration. The hydrophilic surface ensures the uniform distribution of monomers, preventing the formation of defects. By employing Janus substrates fabricated through single-sided deposition of polydopamine/polyethyleneimine, we significantly reduce the thickness of the polyamide nanofilms from 88.4 to 3.8 nm by decreasing the thickness of the hydrophilic layer. This reduction leads to a remarkable enhancement in water permeance from 7.2 to 52.0 l/m^2^·h·bar while still maintaining ~96% Na_2_SO_4_ rejection. The overall performance of this membrane surpasses that of most reported membranes, including state-of-the-art commercial products. The presented strategy is both simple and effective, bringing ultrapermeable polyamide nanofilms one step closer to practical separation applications.

## Introduction

Interfaces offer a confined platform to synthesize two-dimensional (2D) functional materials [1–3]. One of the most notable examples is the interfacial polymerization of polyamide at the liquid–liquid interface, which achieves enormous success in commercial nanofiltration and reverse osmosis membranes for desalination, wastewater recycling, and potable water purification [4–8]. In both industrial and laboratory fabrication processes, interfacial polymerization is normally conducted on porous substrates rather than directly at a free oil–water interface because the nanosized polyamide layers require robust mechanical support to avoid structural failure under high filtration pressure [9,10]. For this reason, the porous substrate serves as an open “interfacial reactor” in this process that not only provides the reaction interface but also reserves and supplies the aqueous monomer solution. On the way to pursuing thin and defect-free polyamide nanofilms with superior separation performance, substantial efforts have been devoted to adjusting the reaction systems for controllable interfacial polymerization [11–20]. Until recently, the vital roles of porous substrates in interfacial polymerization have been gradually recognized, which intrigues escalating research interest in substrate-regulating strategies including surface modification [21], structure regulation [10,22,23], and interlayer construction [24].

Surface wettability and pore structure dominate the impacts of substrates on interfacial polymerization [25,26]. A hydrophilic substrate enables the full spread of the aqueous phase and uniform distribution of monomers on the surface to synthesize thin and defect-free polyamide nanofilms. Beyond directly using hydrophilic materials as substrates, substrate surface engineering strategies such as plasma treatment [27], surface deposition [28,29], and mineralization [30] were also implemented to promote the uniform distribution of monomer solution on the surface, which effectively improved the uniformity of interfacial polymerization and thus endowing the polyamide composite membrane with high performance. On the other hand, the porous structure serves as a reservoir of the monomer solution during interfacial polymerization, while as a mechanical support during filtration. Large-pore substrates have been proven conducive to membrane permeance; however, they need uniform monomer distribution on their surface to ensure the creation of durable polyamide layers capable of withstanding the operation pressure, while the large-pore substrates normally reserve excessive aqueous monomer solution that generates thick polyamide layers with poor water permeance [12,31]. Lowering the monomer concentration is a common strategy to slow down the interfacial polymerization for a thinner polyamide layer, which, however, inevitably compromises the rejection capacity of the membrane due to insufficient cross-linking [13]. Since the pioneering work by Livingston's group [11], extensive research has been conducted on introducing thin sacrificial layers or interlayers as an interfacial reactor to regulate interfacial polymerization [32–34]. Such an interlayer, generally composed of nanoparticles, nanorods, or nanosheets, provides not only a uniform reaction platform but also a reservoir of monomer solution that enables the synthesis of integral polyamide films under a low concentration. However, a sacrificial layer or interlayer may weaken the binding strength between polyamide layers and substrates and is hard to scale up in practical membrane production. Therefore, it is still a challenge to design a substrate with tunable aqueous solution reservation and large pores, which is an ideal platform to achieve thin and dense polyamide layers for both high permeance and satisfying rejection.

Janus membrane refers to a membrane with opposing physicochemical properties, such as the asymmetric wettability on each side, which have found wide uses in multiphase processes such as bubbling, emulsification, demulsification, membrane distillation, and photothermal desalination [35–42]. These membranes generally show superior separation performance by combining the hydrophilic layer and hydrophobic layer to realize directional liquid transport or function integration. One appealing feature of the Janus membranes is their ability to localize the liquid/gas interface within them. This work demonstrates the potential of Janus porous substrates as a powerful tool to regulate interfacial polymerization by their tunable hydrophilic/hydrophobic interface for the first time. Such a structure confines the aqueous solution within a narrow hydrophilic layer, making it possible to conduct interfacial polymerization under a relatively high local monomer concentration but restricted total monomer amount. Here, the Janus substrate is fabricated facilely by single-sided deposition of polydopamine (PDA)/polyethyleneimine (PEI) onto a polypropylene microfiltration membrane (PPMM). The thickness ratio of hydrophilic/hydrophobic layers is tuned by the deposition time. Then, the ultrathin polyamide nanofilms were synthesized by conducting interfacial polymerization on such porous substrates. By tuning the thickness of the hydrophilic layer, the polyamide nanofilm can reach as thin as 3.8 nm, achieving an ultrahigh water permeance of 52 l/m^2^·h·bar and a competitive rejection of 96% to Na_2_SO_4_, which is among the state-of-the-art of polyamide composite membranes.

## Results and Discussion

**Fig. 1.**
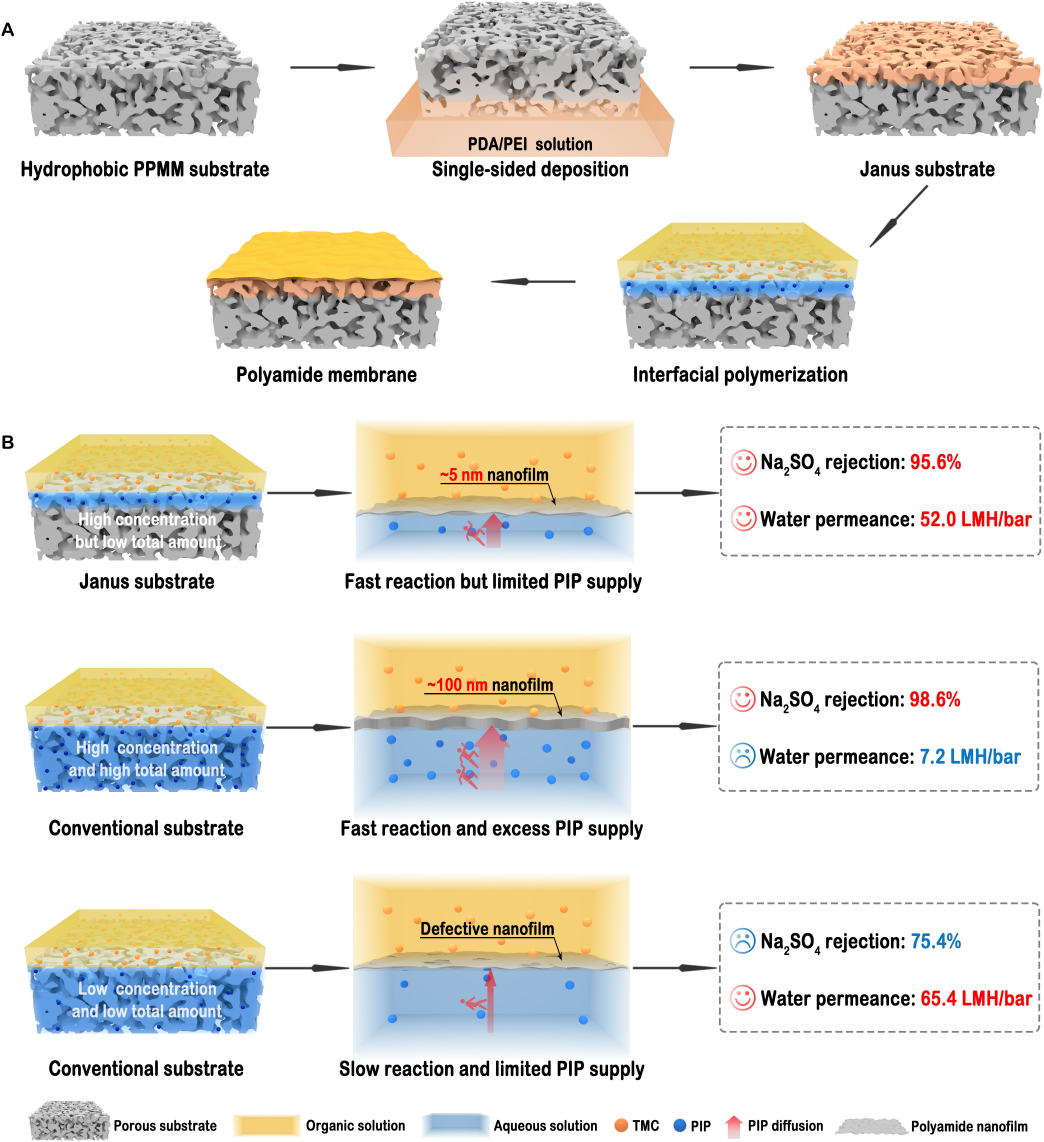
Schematic illustration for synthesizing polyamide nanofilms on different substrates. (A) Fabrication process of polyamide nanofilm on a Janus substrate. (B) Comparison of structure and performance of polyamide nanofilms synthesized on different substrates. Integral and ultrathin nanofilms are synthesized on Janus substrates with high concentration but low amount of diamine monomers, endowing the nanofilms with high rejection performance and excellent water permeance. Conventional hydrophilic substrates result in dense but thick nanofilms at high concentration and high amount of diamine monomers, or thin but defective ones at low concentration and low amount of diamine monomers, resulting in nanofilms with low water permeance or poor rejection performance, respectively.

Figure 1 schematically compares the synthesis processes of polyamide nanofilms on Janus substrate and conventional hydrophilic substrate via interfacial polymerization. Conventional hydrophilic substrates normally result in either dense but thick polyamide nanofilms at high monomer concentration or thin but loose and defective ones at low monomer concentration. By contrast, the Janus substrate owns a tunable hydrophilic layer that can serve as the reservoir of the aqueous solution to control the total amount of amine monomer. Such a structure realizes a high local monomer concentration and low total monomer amount. The high local monomer concentration ensures the formation of a robust and dense nanofilm at the initial stage of interfacial polymerization, and the low total monomer amount allows for achieving a sub–5-nm nanofilm by avoiding the overgrowth of the nanofilm at the later stage of interfacial polymerization, leading to a polyamide composite membrane with both high permeance and selectivity (Fig. 1).

### Fabrication and characterization of Janus substrates

**Fig. 2.**
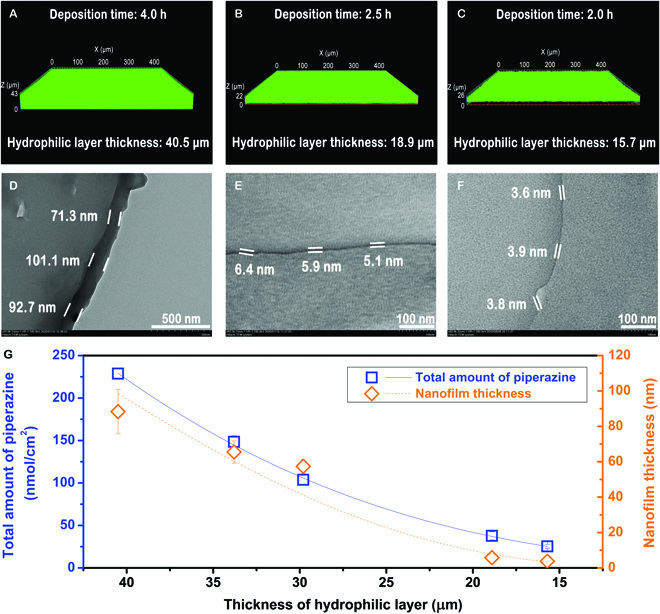
Regulation of nanofilm thickness by Janus substrates. 3D LSCM images of Janus porous substrates absorbed by piperazine solution with deposition time of (A) 2.0 h, (B) 2.5 h, and (C) 4.0 h. Fluorescein sodium was added to the solution to visualize the hydrophilic layer. Cross-sectional TEM images of the polyamide nanofilms synthesized on Janus porous substrates with hydrophilic layer thicknesses of (D) 40.5 μm, (E) 18.9 μm, and (F) 15.7 μm, respectively. (G) Variation of the total amount of piperazine and nanofilm thickness versus the hydrophilic layer thickness of substrates. The concentrations are 34.8 and 22.6 mM for piperazine and trimesoyl chloride used in synthesizing these polyamide nanofilms, respectively.

As mentioned above, the Janus substrate is facilely prepared via single-sided co-deposition of PDA/PEI on PPMM (Figs. S1 and S2). The thickness of the hydrophilic layer is tuned from 2.9 to 40.5 μm with an increased deposition period from 1 to 4 h (Fig. 2A to C and Fig. S3). With the increasing thickness of hydrophilic layer, the absorption capacity of the aqueous diamine solution is adjusted from 0.09 ± 0.01 to 6.57 ± 0.15 mg/cm^2^ (Fig. S4). On the other hand, the initial water contact angle of the deposited surface is reduced below 50° after more than 1 h of deposition, leading to the uniform distribution of the aqueous solution of diamine molecules (Fig. S5). Moreover, no obvious change was observed from the surface morphology of the porous substrate after 4 h of deposition, indicating that the PDA/PEI layer would not affect the substrate permeance (Fig. S1).

### Structures and properties of polyamide nanofilms synthesized on Janus substrates

Interfacial polymerization of piperazine and trimesoyl chloride was conducted on the hydrophilic side of Janus substrates at common monomer concentrations used extensively in industry. The polyamide nanofilm thickness is handily adjusted by the thickness of hydrophilic layers to tune the absorption of diamine solution within substrates (Fig. 2D to G and Fig. S6). The piperazine reserved within the Janus substrate declined from 228.7 to 25.4 nmol/cm^2^ with the same piperazine concentration of 34.8 mM when the thickness of hydrophilic layers decreased from 40.5 to 15.7 μm. Meanwhile, the nanofilm thickness is dramatically reduced from 88.4 to 3.8 nm as the thickness of hydrophilic layers decreases. It is worth noting the polyamide nanofilm with a thickness of 3.8 nm is one of the thinnest polyamide nanofilms as reported in the literature [11–13]. This result demonstrates that Janus substrates provide an accessible, alternative, and novel “interfacial reactor” to effectively regulate the polyamide nanofilm thickness. Moreover, the hydrophobic layer can prevent the penetration of the aqueous solution into the substrate, especially for the large-pore substrates.

The effect of the total piperazine amount on nanofilm morphology was observed by SEM. No polyamide nanofilm is formed on the nascent PPMM substrate because the diamine solution cannot be spread on the hydrophobic surface (Fig. S7). The porous surface was gradually covered by polyamide nanofilms synthesized by an increased diamine amount. The critical amount of piperazine to fabricate an integral polyamide nanofilm on Janus substrate is 25.4 ± 2.3 nmol/cm^2^, which is only about one-ninth of the diamine usage on a conventional hydrophilic substrate [43].

**Fig. 3.**
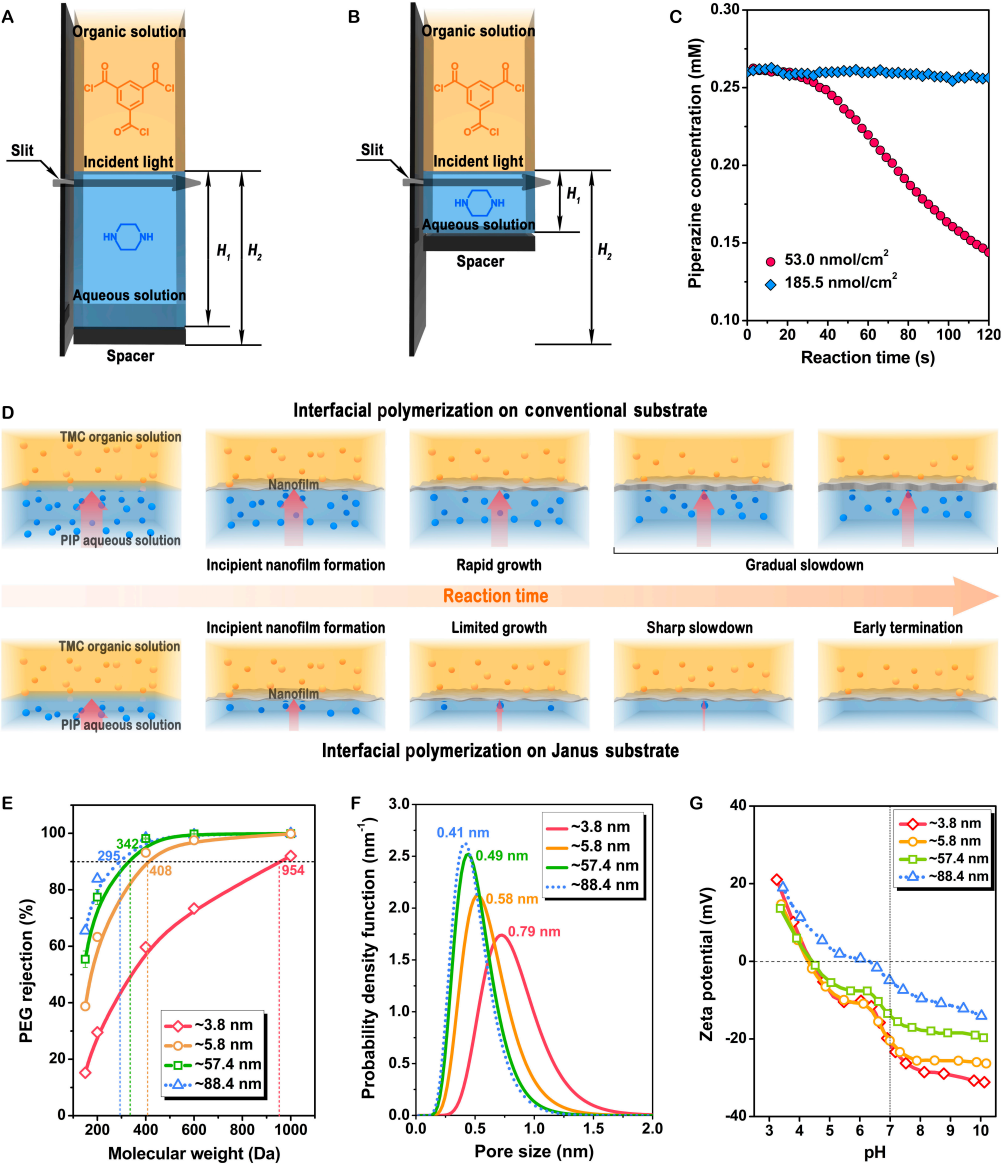
Investigation of interfacial polymerization process and characterization of nanofilm structure and properties. Scheme for illustrating the real-time monitoring of the piperazine concentration change during the interfacial polymerization with (A) high and (B) low total amount of piperazine by UV–vis spectrophotometer. *H*_1_ represents the height of the aqueous solution, and *H*_2_ represents the sum of the height of the spacer and the solution. (C) Concentration change of piperazine during the interfacial polymerization. The concentrations of piperazine and trimesoyl chloride are 0.26 and 18.83 mM, respectively. (D) Schematic illustration of nanofilm growth during interfacial polymerization under different amounts of piperazine. (E) Molecular weight cutoff, (F) pore size distribution, and (G) surface zeta potential of the polyamide nanofilms with different nanofilm thicknesses. The concentrations are 34.8 and 22.6 mM for piperazine and trimesoyl chloride used in synthesizing these polyamide nanofilms, respectively.

The reaction kinetics under varying total diamine monomer amounts were investigated by in situ monitoring of monomer consumption to reveal the underlying regulating mechanisms by the hydrophilic layer thickness (Fig. 3 and Figs. S8 and S9). The absorbance of a charge–transfer complex formed by piperazine and alizarin red at 520 nm linearly increases with the piperazine concentration [15]. Thus, the piperazine consumption can be determined by the absorbance change during interfacial polymerization. A real-time mode was used to monitor the concentration evolution near the reaction interface (Fig. 3A and B). To simulate the role of the hydrophilic layer, the solution volume rather than the diamine concentration was changed to adjust the total monomer amount, and the reaction interface was fixed by applying a spacer for different solution volumes. As shown in Fig. 3C, the absorbance near the interface decreases more sharply with a lower piperazine amount, indicating that the piperazine concentration near the interface decreased more quickly. The reduced concentration may decrease the diffusion rate of piperazine across the interface to the reaction zone according to Fick's law, which can slow down the formation of polyamide nanofilms. Furthermore, by calculating the monomer consumption, lower piperazine participated in the reaction with decreased total amount of piperazine under the same reaction time. This result proves that the aqueous solution volume or total monomer amount control provides an effective way to adjust the reaction kinetics, polymerization window, and polyamide growth.

On the other hand, the reaction kinetics can also be reflected by the consumption rate of trimesoyl chloride, which can be obtained by in situ monitoring its concentration change according to the positive linear relationship between the concentration and the absorbance peak of the organic solution at 290 nm (Figs. S8 and S9). Lower trimesoyl chloride was consumed with decreased total amount of piperazine under the same reaction time, indicating that the participation of trimesoyl chloride in the reaction can also be controlled. These results mean that the reaction between the two monomers can be confined remarkably by limiting the total amount of the diamine monomer solution, which accounts for the reduced thickness of polyamide nanofilms (Fig. 2). According to the monitoring of the reaction kinetics of interfacial polymerization, the regulation mechanism is proposed (Fig. 3D). At the initial stage of the reaction, a high local piperazine concentration can promote the reaction between piperazine and trimesoyl chloride to form an integral nanofilm. Subsequently, as the piperazine is consumed, the total piperazine amount decreases rapidly and the reaction is terminated at a relatively earlier stage of interfacial polymerization, avoiding the further growth of the nanofilm. This mechanism gives us a guideline to control the violent interfacial polymerization and regulate the structure and property of polyamide nanofilms.

The surface chemical composition of polyamide nanofilms was analyzed by x-ray photoelectron spectrometry (XPS) (Fig. S10). The peaks of O 1s and N 1s can reflect the cross-linking and hydrolysis structures on the membrane surface (Table S1). The hydrolysis of trimesoyl chloride is a competitive reaction that converts acyl chloride groups into carboxyl groups, leading to an increase in O/N ratio. The O/N ratio increases with the decrease of nanofilm thickness, indicating that more residual acyl chloride groups were hydrolyzed to carboxyl groups with fewer diamine monomers [11].

We investigated the molecular weight cutoff (MWCO) and pore size distribution of the nanofilms by analyzing the rejection curves of the neutral solutes, as illustrated in Fig. 3E and F. The average pore diameter rises from 0.46 to 0.58 nm as the nanofilm thickness reduces from 88.4 to 5.8 nm. Meanwhile, MWCOs gradually increase from 295 to 408 Da and the pore size distribution is widened slightly. This is because more linear polyamides form when fewer diamines participate in the cross-linking reaction. Significantly, the nanofilm with a thickness of only 5.8 nm still performs a small pore size and narrow pore size distribution. For the ultrathin nanofilm with a thickness of 3.8 nm, the calculated mean pore size dramatically increases to 0.79 nm, demonstrating a loose nanofilm formed when the diamine monomers are insufficient. The XPS spectrum also indicates that more acyl chloride groups were hydrolyzed in the 3.8-nm nanofilm.

In addition to pore size, the surface charge is another crucial factor affecting the separation performance of polyamide nanofilms. The Donnan effect originating from the charged surface plays a crucial role in repelling multivalent ions with the same charges. As the thickness of our nanofilms decreases from 88.4 to 3.8 nm, the surface charge decreases from −4.84 to −21.6 mV at pH 7 (Fig. 3G). This result is also in accordance with the XPS results.

### Nanofiltration performance of polyamide composite membranes

The nanofiltration performance of our polyamide composite membranes was evaluated by a cross-flow filtration apparatus. The effects of diamine monomer amount and concentration on the membrane performance were investigated thoroughly (Fig. S11). The polyamide composite membranes fabricated by piperazine with a common concentration of 34.8 mM and limited total amount show both high water permeance and Na_2_SO_4_ rejection, which is superior to those membranes by the same total monomer amount but lower concentration, or the same concentration but higher total amount (Figs. S11 and S12). The differences in membrane performance stem from the structures of polyamide nanofilms formed under varying reaction conditions. A high concentration and limited amount of piperazine facilitate the formation of ultrathin and integral polyamide nanofilms. In contrast, both a high concentration and amount of piperazine yield dense but thick nanofilms, while a low concentration and limited amount of piperazine result in thin but defective nanofilms. (Fig. 3D and Fig. S13). These results indicate that confining the aqueous solution of amine monomer reserved within the substrates is a more effective way to improve the nanofilm performance than adjusting the diamine concentration. In addition, this confined “interfacial reactor” has the potential to be applied in practical manufacturing because the concentration used in this work has been widely used in industry.

**Fig. 4.**
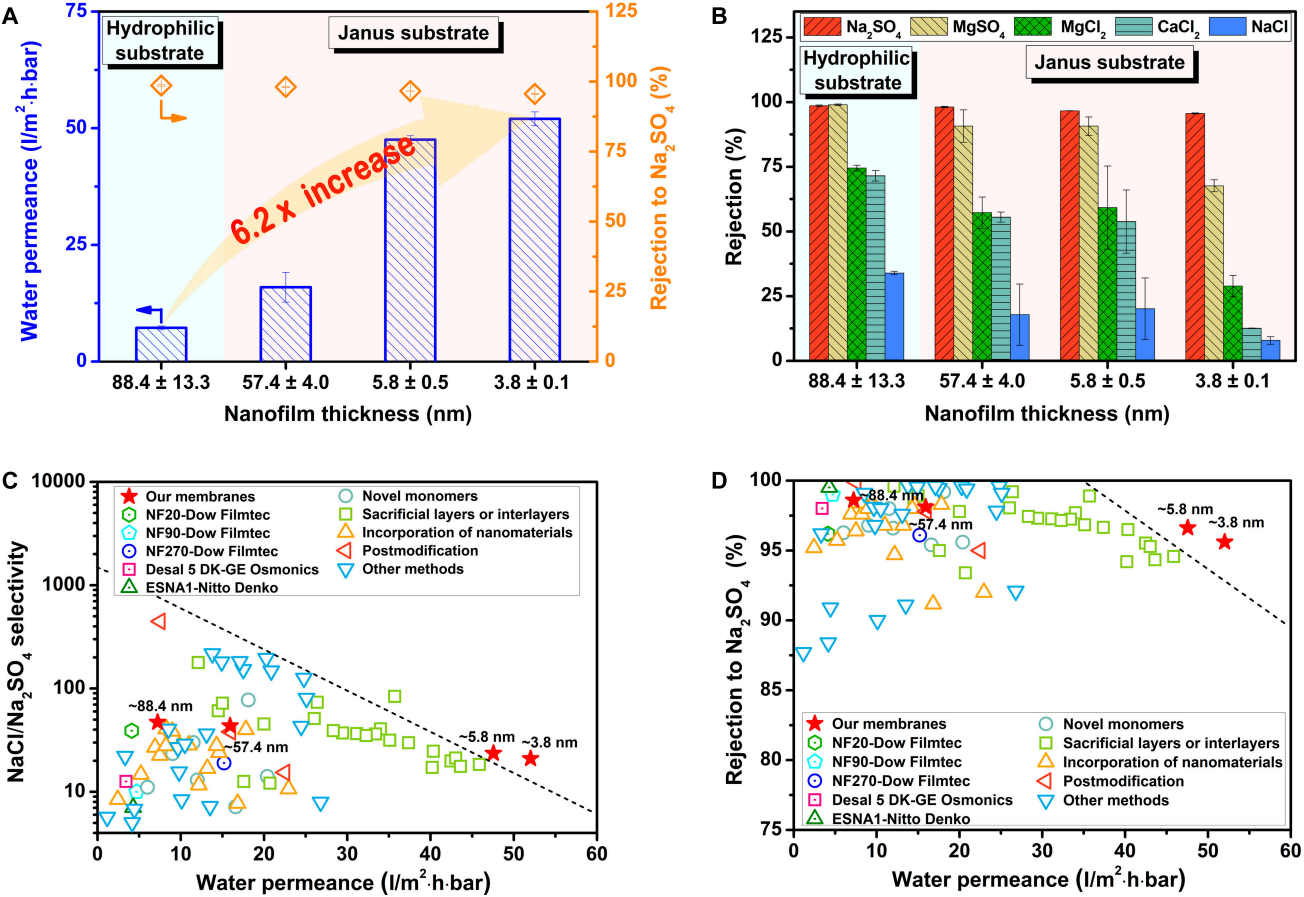
Separation performances of polyamide composite membranes. (A) Rejection performance and water permeance of the polyamide composite membranes with different nanofilm thicknesses for Na_2_SO_4_ solution. (B) Rejection performance of the polyamide composite membranes for various salt solutions. Comparison of (C) the monovalent/divalent salt selectivity and (D) separation performance for Na_2_SO_4_ solution of our membranes and other reported ones. The concentrations are 34.8 and 22.6 mM for piperazine and trimesoyl chloride used in synthesizing these polyamide nanofilms, respectively. The conditions for the performance tests include solute concentrations at 1 g/l, an applied pressure of 4 bar, a cross-flow velocity of 50 l/h, and a temperature of 303 K.

We also studied the effects of nanofilm thickness on the nanofiltration performance of polyamide composite membranes (Fig. 4A). The membranes display a 622% increase in water permeance, soaring from 7.2 to 52.0 l/m^2^·h·bar, as the polyamide nanofilm thickness decreases from 88.4 to 3.8 nm. The ultrathin nanofilms can remarkably reduce transmembrane resistance and facilitate water transport through the composite membranes.

Figure 4B illustrates the salt rejection capacity of the polyamide composite membranes (Fig. S14). On the one hand, the membranes show higher rejection to divalent anions (i.e., ${\mathrm{SO}}_{4}^{2-}$) than monovalent anions (i.e., Cl^−^). Typically, the rejection to Na_2_SO_4_ is 95.6% and 96.6% for the membranes with polyamide nanofilms of 3.8 and 5.8 nm, respectively. On the other hand, the negatively charged surface contributes to the better rejection to divalent anions than divalent cations [44]. As a result, the salt rejection performance of membranes exhibits a sequence of Na_2_SO_4_ > MgSO_4_ > MgCl_2_ > CaCl_2_ > NaCl. Interestingly, the rejection to Na_2_SO_4_ (>95.6%) is much higher than that to NaCl (<34.0%) owing to the stronger electrostatic repulsion and the larger hydrated ion radius of ${\mathrm{SO}}_{4}^{2-}$ (0.38 nm) in contrast to Cl^−^ (0.33 nm) [45], demonstrating excellent separation ratio between monovalent and divalent ions. The NaCl/Na_2_SO_4_ selectivity reaches 21.0 and 23.6 for the composite membranes with nanofilms of 3.8 and 5.8 nm (Fig. 4C). Furthermore, a relatively high rejection to Na_2_SO_4_ (>90%) can be maintained under a wide range of operating pressure from 2 to 8 bar (Fig. S15). On the one hand, the polyamide composite membranes exhibit excellent water permeance of about 50 l/m^2^·h·bar and high Na_2_SO_4_ rejection of about 95% under a low pressure of 2 bar, indicating their great potential in low-pressure nanofiltration. On the other hand, the membranes show decent separation performance with a high Na_2_SO_4_ rejection of above 90% even under high pressure of 8 bar, demonstrating their good mechanical strength during nanofiltration. The robust structure can be ascribed to the high Young's modulus of polyamide nanofilms and the high binding strength between the nanofilm and substrate [11,31,46].

Figure 4D compares the nanofiltration performance of our membranes with other reported nanofiltration membranes (Table S2). Only a few membranes exhibit a water flux above 40 l/m^2^·h·bar while keeping a satisfied Na_2_SO_4_ rejection above 95%. The high-performance membranes are mainly prepared by complex methods, such as the design of novel monomers, incorporation of nanomaterials in selective layers, construction of the sacrificial layers or interlayers, and postmodification of the selective layers. Our membranes have a significant breakthrough in nanofiltration performance when the thicknesses of the polyamide nanofilms are reduced to 5.8 and 3.8 nm, exceeding the performance of most commercial and recently reported nanofiltration membranes. More importantly, the fabrication of our membranes is based on a simple regulation mechanism and a facile preparation process, which can be readily realized by utilizing Janus porous substrates as the “interfacial reactor” of interfacial polymerization for enhancing reaction controllability.

## Conclusion

In summary, we have developed a novel strategy to synthesize ultrathin polyamide nanofilms by virtue of Janus substrates as a confined interfacial polymerization platform to govern the reaction process. The aqueous solution of amine monomer can be confined within the adjustable hydrophilic layer of the Janus substrate, by which the total monomer amount can be regulated for controlling the interfacial polymerization. By tuning the piperazine amount reserved within the Janus substrate at a low level, sub–5-nm polyamide nanofilms have been synthesized at a relatively high local piperazine concentration, which endows the polyamide nanofilms with an integral structure and small pore size. The water permeance has greatly elevated by more than six times when the nanofilm thickness decreases from 88.4 to 3.8 nm while keeping a competitive Na_2_SO_4_ rejection of ~96%. Such a composite membrane performs superior nanofiltration performance to most of the recently reported ones. This strategy offers a novel and practical “interfacial reactor” to regulate the interfacial polymerization process as well as the nanofilm structure by engineering the porous substrates.

## Materials and Methods

### Materials and chemical reagents

Materials and chemical reagents can be found in the Supplementary Materials (Section S1.1).

### Fabrication of Janus substrates by single-sided co-deposition

The Janus porous substrates were prepared by single-sided co-deposition of PDA/PEI on PPMM [38]. A solution of dopamine (DA) and PEI (2.0 mg/ml) was prepared by dissolving them into a tris buffer solution (pH 8.5, 50.0 mM). The nascent PPMM sample was pre-wetted with ethanol and placed on DA/PEI solution under ambient temperature for 1 to 4 h. Then, the samples were cleaned by ultrapure water and dried under vacuum.

### Measurements of the hydrophilic layer thickness and the piperazine absorption capacity of Janus substrates

The Janus substrate was floated on a 0.01 wt % sodium fluorescein solution with the hydrophilic side downward for 5 min. Then, a laser scanning confocal microscope (LSCM; Zeiss LSM780, Germany) was used to observe the substrate. The hydrophilic layer thickness was determined by measuring the luminous portion of the substrate according to the green fluorescence of fluorescein sodium in the aqueous solution under 488-nm exciting light.

Similarly, Janus substrates with an area of 19.6 cm^2^ were floated on the piperazine aqueous solution (34.8 mM) to absorb the piperazine solution. The redundant liquid on the substrate surface was wiped off. The substrates before and after absorbing the piperazine solution were weighed to calculate their storage capacity.

### Interfacial polymerization on Janus substrates

The polyamide nanofilm was created on the Janus substrate via the interfacial polymerization of piperazine and trimesoyl chloride. Piperazine solution was prepared with concentrations of 11.6, 23.2, and 34.8 mM by dissolving it in water. The solution of trimesoyl chloride was prepared with concentrations of 7.5, 15.1, and 22.6 mM by dissolving it in Isopar H. First, the Janus substrate was floated on piperazine solution with the hydrophilic side downward. The porous substrate was picked up after 5 min, and the redundant liquid on the hydrophilic side was removed. Then, 2 ml of trimesoyl chloride solution was added to the hydrophilic surface to trigger the reaction. The trimesoyl chloride solution was removed after 2 min. Afterward, the samples were dried under 100 °C. After 5 min, the prepared membranes were reserved in water for subsequent characterization and tests.

### Characterization of Janus substrates and polyamide composite membranes

The chemical composition of the surface of the Janus substrate and composite membrane was characterized by XPS (Thermo Fisher Scientific, K-Alpha+, USA) using Al Kα excitation radiation (1,486.6 eV). The membranes were rinsed with pure water, dried in a vacuum oven, and cut into 5 mm × 5 mm samples for the XPS characterization. The polyamide network consists of cross-linked part and linear part. The cross-linking degree (*DC*) is defined as the ratio of the cross-linked structure to the entire structure [47]. The *DC* of the polyamide nanofilms was calculated by the equations [11]:

$$DC=\frac{A}{A+B}\;$$ (1)

$$\frac{O}{N}=\frac{3A+4B}{3A+2B}$$ (2)

where *O*/*N* indicates the relative amounts of oxygen to nitrogen, obtained from analyzing the XPS results. *A* and *B* represent the proportion of the cross-linked portion and linear portion, respectively.

The cross-sectional and surface morphology of the Janus porous substrate and the polyamide composite membrane were investigated using field emission scanning electron microscopy (FESEM; Hitachi, S4800) under 3 kV. The samples were rinsed with pure water, dried in a vacuum oven, affixed onto the sample stage with conductive adhesive, and sputtered with a platinum layer. In order to observe the cross-sectional morphology of samples, they were brittle-fractured using liquid nitrogen and then attached to the sample stage with the observation surface facing upward. The thickness of the polyamide nanofilms was measured by transmission electron microscopy (TEM; Hitachi, HT-7700, Japan) under 100 kV. The samples were embedded in epoxy resin and then cut by an Ultramicrotome (Leica, EM UC7, Germany). The surface charge of membranes was analyzed using an electrokinetic analyzer (SurPASS Anton Paar, GmbH, Austria) according to a streaming-potential method [48].

Dynamic water contact angles were investigated using a DropMeter A-200 contact angle system (MAIST Vision Inspection & Measurement Co. Ltd., China).

MWCO was obtained by fitting the rejection of electrically neutral polyethylene glycol (PEG) molecules [49,50]. The pore size distribution of the polyamide composite membranes was analyzed by the PEG rejection [43]. The calculation processes are described in the Supplementary Materials.

### In situ monitoring of the reaction process of interfacial polymerization

Monomer concentrations were in situ monitored by an ultraviolet–visible (UV–vis) spectrophotometer (Shimadzu UV2450, Japan) during interfacial polymerization [15]. The piperazine concentration in the aqueous solution and the concentration of trimesoyl chloride in the organic solution were determined according to their linear relationship with the solution absorbance [15]. The concentration evolution reflected the consumption of monomers during interfacial polymerization. The measurement point is 30 μm below or above the reaction interface. A silicon spacer was applied to adjust the reaction interface with different volumes of the aqueous solution. The experimental details are described in the Supplementary Materials.

### Assessment of the nanofiltration performances

The nanofiltration performances of the membranes were evaluated using a cross-flow filtration apparatus. The feed solutions were prepared at a concentration of 1.0 g/l by adding organic salts to ultrapure water. The water flux and salt rejection of the samples were investigated at 4 bar, 30 °C and a cross-flow rate of 50 l/h after pre-compacting for 0.5 h. The following equations were used to calculate the water flux (*F*, l/m⋅h) and water permeance (*P*_1_, l/m⋅h⋅bar):

$$F=\frac{V}{A∙T}$$ (3)

$$P_{1}=\frac{F}{P}$$ (4)

where *V* (l), *A* (m^2^), *T* (h), and *P* (bar) represent the filtrate volume, the filtration area, the testing time, and the operation pressure, respectively. The salt rejection rate (*R*, %) was calculated according to Eq. S6. Solute concentrations were measured using an electrical conductivity meter (Mettler Toledo, FE30, China). All samples underwent testing three times using identical operating conditions.

The solute selectivity (*S*) is determined by comparing the permeation of solutes across the membranes. The selectivity of two solutes can be calculated by the following equation [51]:

$$S=\frac{1-R_{A}}{1-R_{B}}$$ (5)

where *R*_*A*_ and *R*_*B*_ represent the rejection of solutes A and B, respectively.

## Data Availability

The authors declare that the data supporting the findings of this study are available within the paper and its supplementary materials files or available from the corresponding author upon request.

## Supplementary Materials

Supplementary 1 Materials and Methods Figs. S1 to S15 Tables S1 and S2 References [52–96]
