# Supplementary material for: Leveraging Janus Substrates as a Confined “Interfacial Reactor” to Synthesize Ultrapermeable Polyamide Nanofilms
Source: Research (Wash D C). 2024 Apr 29;7:0359. doi: 10.34133/research.0359 (PMC11062503; doi:10.34133/research.0359)
Supplement: Supplementary 1 — Materials and Methods Figs. S1 to S15 Tables S1 and S2 References [52–96] [file research.0359.f1.docx]

**Supplementary Materials**

**Leveraging Janus Substrates as a Confined “Interfacial Reactor” to Synthesize Ultra-permeable Polyamide Nanofilms**

Cheng-Ye Zhu^1,2^, Hao-Nan Li^1^, Bian-Bian Guo^1^, Yu Fang^1^, Chang Liu^1,2^, Hao-Cheng Yang^1,2,^*, Chao Zhang^1,2^, Hong-Qing Liang^1,2^, Zhi-Kang Xu^1,2,^*

^1^ MOE Key Lab of Macromolecular Synthesis and Functionalization, and Key Lab of Adsorption and Separation Materials & Technologies of Zhejiang Province, Department of Polymer Science and Engineering, Zhejiang University, Hangzhou 310058, China.

^2^ The “Belt and Road” Sino-Portugal Joint Lab on Advanced Materials, International Research Center for X Polymers, Zhejiang University, Hangzhou 310058, China.

* Address correspondence to: Hao-Cheng Yang; yanghch@zju.edu.cn and Zhi-Kang Xu; xuzk@zju.edu.cn

**Table of contents**

1. Supplementary Materials and Methods

1.1 Supplementary Materials……………………………………………………………...4

1.2 Characterization of the pore structure of polyamide composite membranes…….…...4

1.3 In situ monitoring of the reaction process of interfacial polymerization………….….5

2. Supplementary Figures

Fig. S1. SEM images of the (a) nascent polypropylene microfiltration membrane surface and (b-f) polydopamine/polyethyleneimine-modified surfaces of Janus membranes with various deposition time………………...……………………………………………….…7

Fig. S2. Effect of the deposition time on the deposition weight of polydopamine/polyethyleneimine…………………………………………………………8

Fig. S3. 3D LSCM images of Janus membranes absorbed by piperazine solution containing fluorescein sodium with various deposition time………………….…9

Fig. S4. Effect of deposition time on the absorption capacity of Janus membranes for piperazine solution…………………………………….…………………………………10

Fig. S5. Dynamic water contact angles on the polydopamine/polyethyleneimine-modified surfaces of Janus membranes with various deposition time………………………………..………...……………………………………………11

Fig. S6. Cross-sectional TEM images of the polyamide nanofilms synthesized on Janus substrates with different total amounts of piperazine via interfacial polymerization…………………………………………………………………………...12

Fig. S7. SEM images of the polyamide nanofilms synthesized on Janus substrates with different total amounts of piperazine via interfacial polymerization………………….…13

Fig. S8. Schematic illustration for real-time monitoring of the concentration change of (a) piperazine and (b) trimesoyl chloride during the interfacial polymerization by UV-vis spectrophotometer………………………………………………………...……………...14

Fig. S9. Concentration change of (a) piperazine in the aqueous solution and (b) trimesoyl chloride in the organic solution during the interfacial polymerization……15

Fig. S10. XPS survey spectra of the nascent polypropylene microfiltration membrane, polydopamine/polyethyleneimine-modified membrane and polyamide nanofilms synthesized on the Janus substrates with different total amounts of piperazine via interfacial polymerization…………………………………………………….……...16

Fig. S11. Nanofiltration performances of the polyamide composite membranes for Na_2_SO_4_ solution…………………………….………………………………...………….17

Fig. S12. Nanofiltration performances of the polyamide composite membranes for Na_2_SO_4_ solution……………………….……………………………...………………….18

Fig. S13. (a) Molecular weight cut-off and (b) pore size distribution of the polyamide nanofilms synthesized on hydrophilic porous substrates via interfacial polymerization…………………………………………………………………...………19

Fig. S14. Rejection performance of the polyamide composite membranes for different inorganic salt aqueous solutions…………………………………………………...…….20

3. Supplementary Tables

Table S1. Atomic composition of the nascent polypropylene microporous membrane and polydopamine/polyethyleneimine-modified membrane………………….……………21

Table S2. Comparison of the nanofiltration performance between the as-prepared and the recently reported nanofiltration membranes……………………………………….…….22

4. Supplementary References……………………………………………..…….…………….25

**1. Supplementary Materials and Methods**

**1.1 Supplementary Materials**

Polypropylene microfiltration membranes (PPMM, average pore size = 0.2 μm) were a commercial product from Membrana GmbH (Germany). The membranes were rinsed with acetone for 5 h to remove the adsorbed impurities and dried in a vacuum oven at room temperature overnight. Dopamine hydrochloride (DA) was bought from Sigma-Aldrich (USA). Polyethyleneimine (PEI, M_w_ = 600 Da), piperazine (99.0%) and alizarin red were purchased from Aladdin Chemical Co., Ltd. (China). Trimesoyl chloride (99.0%) was obtained from Qingdao Benzo Chemical Co., Ltd. (China). Isopar H was supplied by Sharun Chemical Co., Ltd. (China). Fluorescein sodium was obtained from Macklin Biochemical Co., Ltd. (China). Other chemicals, including hydrochloric acid (HCl) solution (12.0 M), sodium hydroxide (NaOH), potassium hydroxide (KOH), sodium sulfate (Na_2_SO_4_), magnesium sulfate (MgSO_4_), magnesium chloride (MgCl_2_), calcium chloride (CaCl_2_), sodium chloride (NaCl), triethylene glycol and polyethylene glycols (PEG) with various molecular weights, were purchased from Sinopharm Chemical Reagent Co., Ltd (China) and utilized as received without further purification. Ultrapure water (18.2 MΩ) was produced from a lab water purification system (ELGA Lab Water system, France).

**1.2** **Characterization of the pore structure of polyamide composite membranes**

The molecular weight cut-off (MWCO) and pore size were calculated by fitting the rejection of electrically neutral solutes with different molecular weights. Triethylene glycol (150 Da) and PEG (200 Da, 400 Da, 600 Da and 1000 Da) solutions with a concentration of 1.0 g/L were applied to detect the rejection by a cross-flow filtration device^[40]^. The solute rejection (*R*, %) was calculated by the following equation:

$$R=\left( \text{1}-\frac{C_{p}}{C_{f}} \right)\times\text{100}\% \text{(}\text{6}\text{)}$$

where *C_p_* and *C_f_* represent the solute concentrations in the filtrate and feed respectively, which were measured with a total organic carbon analyzer (TOC, GE Sievers InnovOx ES, USA). MWCO is assigned as the molecular weight of the solute with a rejection rate of 90%^[41]^.

The pore size distribution of the polyamide composite membranes was analyzed by the PEG rejection. The mean pore size (*μ*_p_) is equal to the solute size (*d*_s_) with a rejection of 50% if the steric and hydrodynamic interactions between solutes and membrane pores were ignored. And the geometric standard deviation (*σ*_p_) is defined as the rate of *d*_s_ with a rejection rate of 84.13% and 50%. The Stokes radius (*r*_s_, nm) of the solute can be obtained from the next equation^[40]^:

$$r_{s}=\text{16.73}\times\text{10}^{-\text{3}}M^{\text{0.557}} \text{(}\text{7}\text{)}$$

where *M* is the molecular weight of PEG.

The pore size distribution of the membranes is calculated by the following formula^[40]^:

$$\frac{df\left( d_{p} \right)}{dd_{p}}=\frac{\text{1}}{d_{p}\ln\sigma_{p}\sqrt{\text{2π}}}\exp\left[ -\frac{\left( \ln d_{p}-\ln\mu_{p} \right)^{\text{2}}}{{\text{2}\left( \ln\sigma_{p} \right)}^{\text{2}}} \right] \text{(}\text{8}\text{)}$$

where *d*_p_ is the pore diameter.

**1.3** **In situ monitoring of the reaction process of interfacial polymerization**

The monomer concentrations were in situ monitored by an ultraviolet-visible (UV-vis) spectrophotometer (Shimadzu UV2450, Japan) during interfacial polymerization^[15]^. The piperazine concentration in the aqueous solution was measured as follows. Piperazine and alizarin red were dissolved in ultrapure water with a concentration of 0.26 mM and 0.71 mM, respectively. Trimesoyl chloride was dissolved in Isopar H with a concentration of 18.83 mM. A certain amount of the piperazine solution was added into a cuvette with a bottom edge of 1 cm. Then, 0.55 mL trimesoyl chloride solution was gently added onto the piperazine solution to trigger the interfacial polymerization. The absorbance change of the aqueous solution at 520 nm was immediately measured by the UV-vis spectrophotometer through a slit on the sample holder.

The concentration of trimesoyl chloride in the organic solution was measured as follows. Piperazine was dissolved in ultrapure water with a concentration of 2.32 mM. Trimesoyl chloride was dissolved in Isopar H with a concentration of 0.38 mM. A certain amount of the piperazine solution was added into a cuvette and 0.40 mL trimesoyl chloride solution was gently added onto the surface of the piperazine solution to trigger the interfacial polymerization. The absorbance evolution of the organic solution at 290 nm was immediately measured by the UV-vis spectrophotometer through a slit on the sample holder.

The measurement point is 30 μm below or above the reaction interface. A silicon spacer was applied to adjust the reaction interface with different volumes of the aqueous solution. The monomer concentrations were determined according to their linear relationship with the solution absorbance^[15]^, and the concentration evolution reflected the consumption of monomers during interfacial polymerization.

**2. Supplementary Figures**


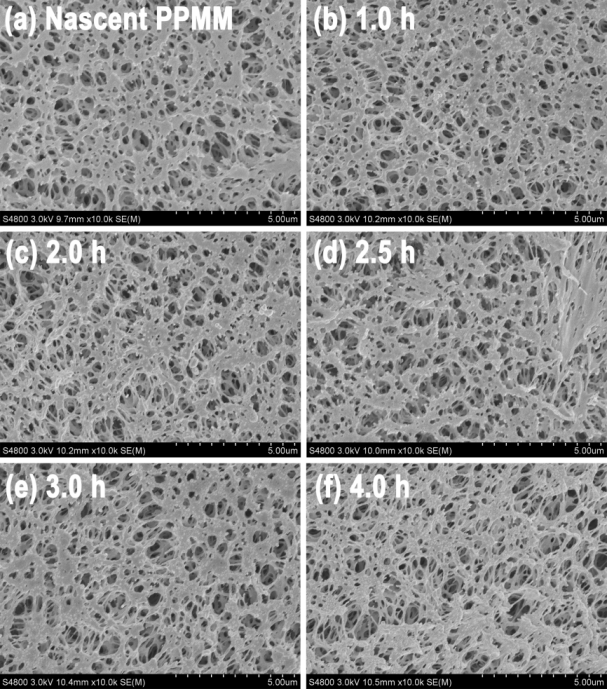


**Fig. S1.** SEM images of the (a) nascent polypropylene microfiltration membrane surface and (b-f) polydopamine/polyethyleneimine-modified surfaces of Janus membranes with various deposition time. Both polydopamine and polyethyleneimine were dissolved into the Tris buffer solution (pH = 8.5, 50.0 mmol/L) with a concentration of 2.0 mg/mL.

**Fig. S2.** Effect of the deposition time on the deposition weight of polydopamine/polyethyleneimine. Both polydopamine and polyethyleneimine were dissolved into the Tris buffer solution (pH = 8.5, 50 mmol/L) with a concentration of 2.0 mg/mL.


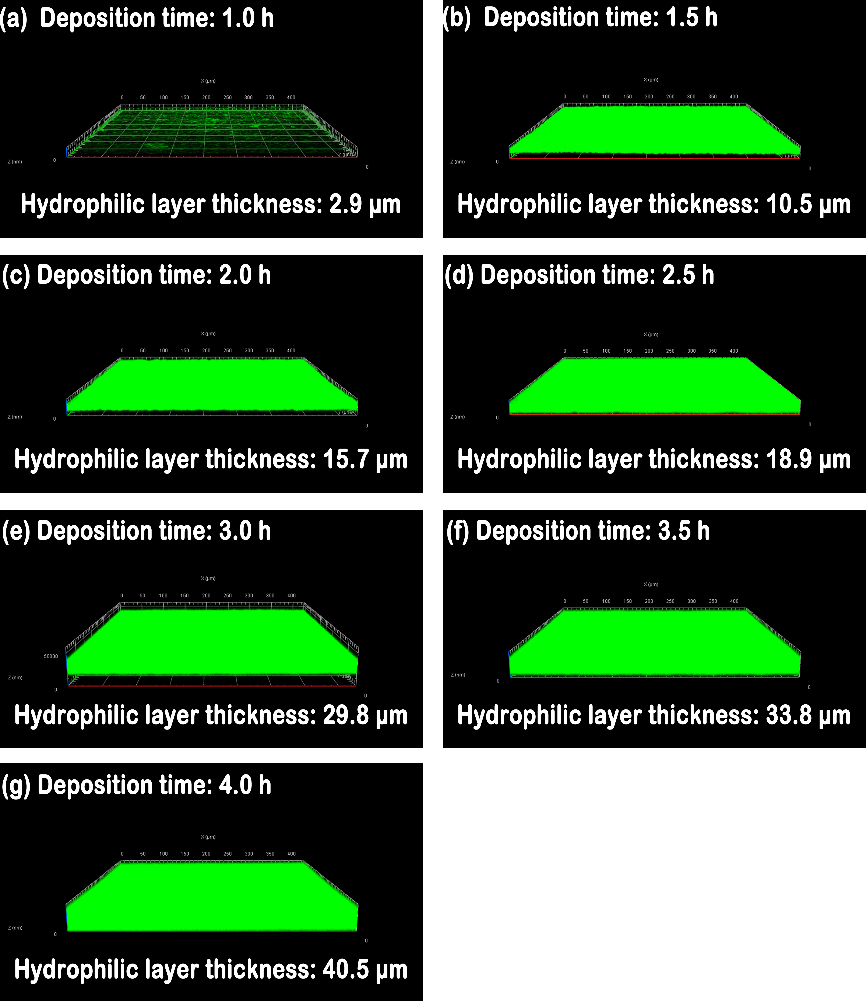


**Fig. S3.** 3D LSCM images of Janus membranes absorbed by piperazine solution containing fluorescein sodium with various deposition time. Both polydopamine and polyethyleneimine were dissolved into the Tris buffer solution (pH = 8.5, 50.0 mmol/L) with a concentration of 2.0 mg/mL.

**Fig. S4.** Effect of deposition time on the absorption capacity of Janus membranes for piperazine solution. Both polydopamine and polyethyleneimine were dissolved into the Tris buffer solution (pH = 8.5, 50.0 mmol/L) with a concentration of 2.0 mg/mL.

**Fig. S5.** Dynamic water contact angles on the polydopamine/polyethyleneimine-modified surfaces of Janus membranes with various deposition time. Both polydopamine and polyethyleneimine were dissolved into the Tris buffer solution (pH = 8.5, 50.0 mmol/L) with a concentration of 2.0 mg/mL.


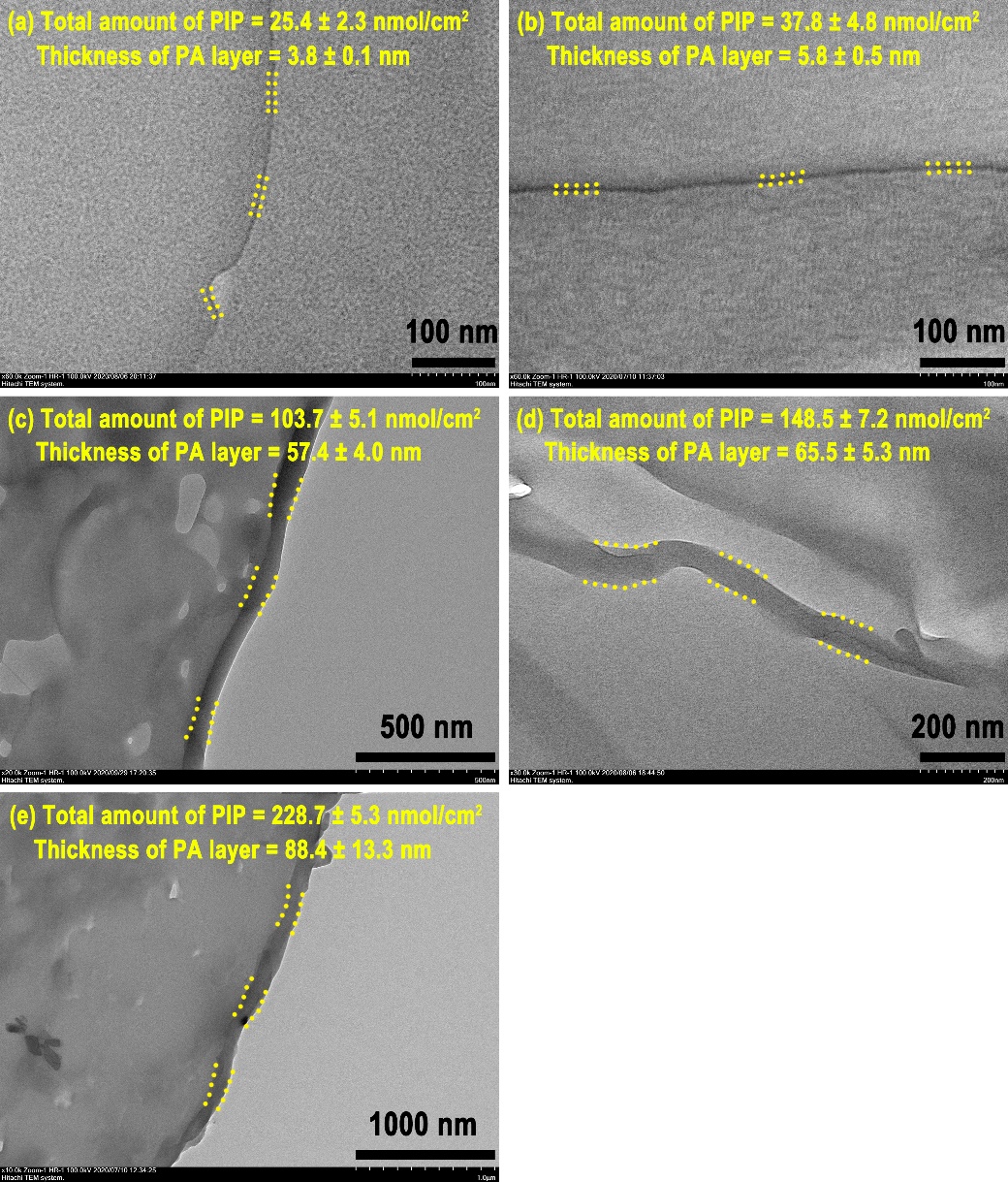


**Fig. S6.** Cross-sectional TEM images of the polyamide nanofilms synthesized on Janus substrates with different total amounts of piperazine via interfacial polymerization. The concentrations of piperazine and trimesoyl chloride are 34.8 mM and 22.6 mM, respectively. The reaction time and thermal treatment time are 120 s and 5 min, respectively.


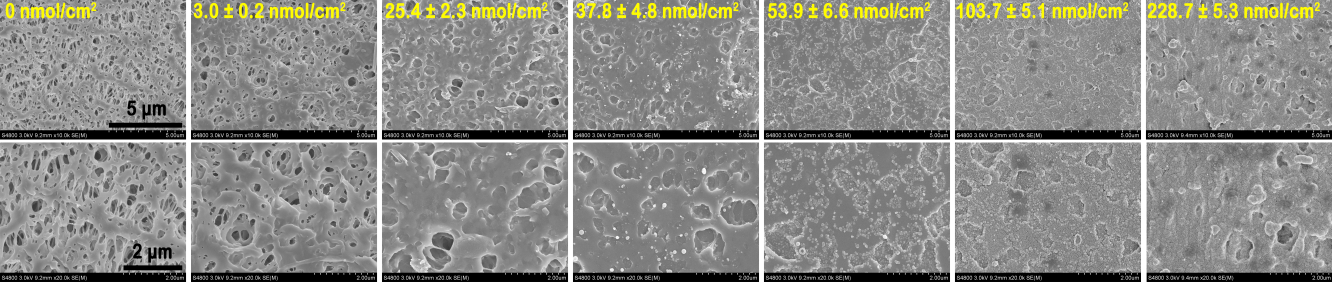


### **Fig. S7.** SEM images of the polyamide nanofilms synthesized on Janus substrates with different total amounts of piperazine via interfacial polymerization. The concentrations of piperazine and trimesoyl chloride are 34.8 mM and 22.6 mM, respectively. The reaction time and thermal treatment time are 120 s and 5 min, respectively.

**
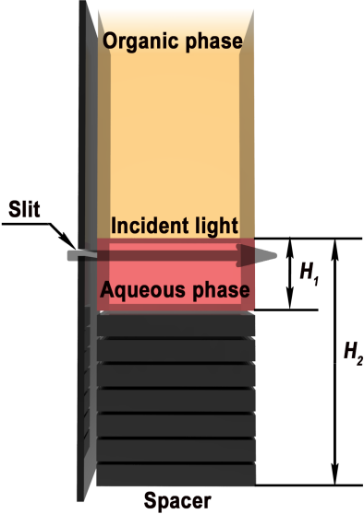
** **
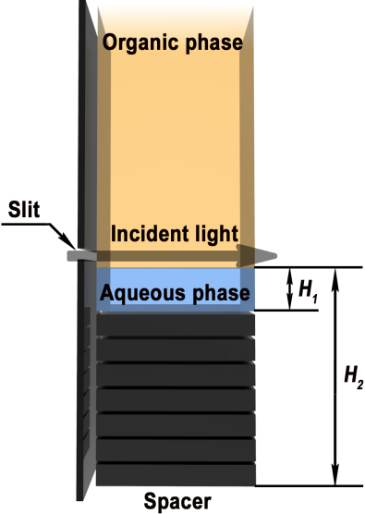
**

**(b)**

**(a)**

**Fig. S8.** Schematic illustration for real-time monitoring of the concentration change of (a) piperazine and (b) trimesoyl chloride during the interfacial polymerization by UV-vis spectrophotometer. *H_1_* represents the height of the aqueous solution and *H_2_* represents the sum of the height of the spacer and the height of the aqueous solution.

**(b)**

**(a)**

**(d)**

**(c)**

**Fig. S9.** Concentration change of (a) piperazine in the aqueous solution and (b) trimesoyl chloride in the organic solution during the interfacial polymerization. Effect of the total amount of piperazine on the consumption of (c) piperazine and (d) trimesoyl chloride during the interfacial polymerization. For (a) and (c), the concentrations of piperazine and trimesoyl chloride are 0.26 mM and 18.83 mM, respectively, and the concentration of alizarin red is 0.71 mM. For (b) and (d), the concentrations of piperazine and trimesoyl chloride are 2.32 mM and 0.38 mM, respectively.

**Fig. S10.** XPS survey spectra of the nascent polypropylene microfiltration membrane, polydopamine/polyethyleneimine-modified membrane and polyamide nanofilms synthesized on the Janus substrates with different total amounts of piperazine via interfacial polymerization. The deposition time is 2.5 h for the polydopamine/polyethyleneimine-modified membrane. The concentrations are 34.8 mM and 22.6 mM for piperazine and trimesoyl chloride used in the synthesis of these polyamide nanofilms, respectively. The interfacial polymerization time and thermal treatment time are 120 s and 5 min, respectively. PA-X represents that the thickness of the polyamide nanofilm is X nm.

**(a)**

**(b)**

**(c)**

**Fig. S11.** Nanofiltration performances of the polyamide composite membranes for Na_2_SO_4_ solution. The polyamide nanofilms of the composite membranes were synthesized with different absorption capacity of piperazine solution via interfacial polymerization. The concentrations of piperazine and trimesoyl chloride are (a) 11.6 mM and 7.5 mM, (b) 23.2 mM and 15.1 mM, (c) 34.8 mM and 22.6 mM, respectively. The reaction time and thermal treatment time are 120 s and 5 min, respectively. Test conditions: concentration of Na_2_SO_4_ = 1 g/L, temperature = 303 K, pressure = 4 bar, and cross-flow rate = 50 L/h.

**(b)**

**(a)**

**Fig. S12.** Nanofiltration performances of the polyamide composite membranes for Na_2_SO_4_ solution. The polyamide nanofilms of the composite membranes were synthesized with similar total amounts but different concentrations of piperazine. For (a), the total amount of piperazine is around 25 nmol/cm^2^. The concentrations of piperazine are 3.8 mM, 5.0 mM, and 34.8 mM. The concentration of trimesoyl chloride is 22.6 mM. For (b), the total amount of piperazine is around 36 nmol/cm^2^. When the concentrations of piperazine are 11.6 mM, 23.2 mM, and 34.8 mM, the concentrations of trimesoyl chloride are 7.5 mM, 15.1 mM, and 22.6 mM, respectively. The reaction time and thermal treatment time are 120 s and 5 min, respectively. Test conditions: concentration of Na_2_SO_4_ = 1 g/L, temperature = 303 K, pressure = 4 bar, and cross-flow rate = 50 L/h.

**(b)**

**(a)**

**Fig. S13.** (a) Molecular weight cut-off and (b) pore size distribution of the polyamide nanofilms synthesized on hydrophilic porous substrates via interfacial polymerization. The total amount of piperazine is around 25 nmol/cm^2^. The concentrations of piperazine and trimesoyl chloride are 4.1 mM and 22.6 mM, respectively. The reaction time and thermal treatment time are 120 s and 5 min, respectively. Testing conditions: concentrations of the solutes in the testing solutions = 1.0 g/L, applied pressure = 0.4 MPa, cross-flow velocity = 50.0 L/h, and temperature = 303 K.

**Fig. S14.** Rejection performance of the polyamide composite membranes for different inorganic salt aqueous solutions. The polyamide nanofilms of the composite membranes were synthesized on Janus porous substrates via interfacial polymerization. The concentrations of piperazine and trimesoyl chloride are 34.8 mM and 22.6 mM, respectively. The reaction time and thermal treatment time are 120 s and 5 min, respectively. Testing conditions: concentrations of the solutes in the testing solutions = 1 g/L, applied pressure = 4 bar, cross-flow velocity = 50 L/h, and temperature = 303 K.

**Fig. S15.** Rejection performance and water permeance of the polyamide composite membranes under different operating pressures for Na_2_SO_4_ solution. The conditions for the performance tests include Na_2_SO_4_ concentration at 1 g/L, a cross-flow velocity of 50 L/h, and a temperature of 303 K.

**3. Supplementary Tables**

**Table S1.** Atomic composition of the nascent polypropylene microporous membrane and polydopamine/polyethyleneimine-modified membrane. Total amount of diamine monomers, atomic composition, O/N ratio and cross-linking degree of the polyamide nanofilms synthesized with different total amounts of piperazine via interfacial polymerization.

| Sample^a)^ | Total amount of piperazine (nmol/cm^2^) | Atomic composition from XPS^b)^ | | | O/N ratio | Cross-linking degree^c)^  (%) |
| --- | --- | --- | --- | --- | --- | --- |
|  |  | C | N | O |  |  |
| PPMM | — | 99.37 | 0.23 | 0.39 | — | — |
| PPMM-PDA/PEI | — | 77.24 | 7.99 | 14.77 | 1.85 | — |
| PA-3.8^d)^ | 25.4 ± 2.3 | 68.47 | 8.42 | 23.11 | 2.74 | — |
| PA-5.8 | 37.8 ± 4.8 | 70.33 | 10.71 | 18.96 | 1.77 | 16.6 |
| PA-57.4 | 103.7 ± 5.1 | 70.53 | 11.23 | 18.24 | 1.62 | 28.6 |
| PA-88.4 | 228.7 ± 5.3 | 70.58 | 14.14 | 15.27 | 1.08 | 88.5 |

^a)^The polyamide nanofilms were synthesized with concentrations of 34.8 mM and 22.6 mM for piperazine and trimesoyl chloride, respectively. The interfacial polymerization time and thermal treatment time are 120 s and 5 min, respectively. ^b)^The element composition was assessed by XPS measurement. ^c)^The cross-linking degree was calculated from the ratio of the fully cross-linked structure in the whole polyamide structure. ^d)^PA-X represents that the thickness of the polyamide nanofilm is X nm.

**Table S2.** Comparison of the nanofiltration performance between the as-prepared and the recently reported nanofiltration membranes.

| Ref. | Rejection to Na_2_SO_4_ (%) | Rejection to NaCl (%) | NaCl/Na_2_SO_4_ selectivity | Water permeability (L/ m^2^⋅h⋅bar) | Salt concentration (g/L) | Operating pressure (bar) |
| --- | --- | --- | --- | --- | --- | --- |
| This work | | | | | | |
| PA-88.4 nm^a)^ | 98.60 | 33.96 | 47.17 | 7.22 | 1 | 4 |
| PA-57.4 nm | 98.11 | 17.84 | 43.54 | 15.92 | 1 | 4 |
| PA-5.8 nm | 96.62 | 20.16 | 23.61 | 47.56 | 1 | 4 |
| PA-3.8 nm | 95.61 | 7.90 | 20.96 | 52.02 | 1 | 4 |
| Novel monomers | | | | | | |
| [52] | 95.4 | 67.0 | 7.2 | 16.6 | 1 | 6 |
| [53] | 95.6 | 38.0 | 14.1 | 20.4 | 1 | 3 |
| [54] | 96.3 | 58.7 | 11.1 | 6.0 | 1 | 5 |
| [55] | 96.6 | 55.7 | 13.0 | 12.0 | 2 | 6 |
| [56] | 96.8 | 25.0 | 23.4 | 9.0 | 2 | 4 |
| [57] | 98.0 | 40.0 | 30.0 | 11.5 | 10 mM | 4 |
| [58] | 99.2 | 38.0 | 77.5 | 18.1 | 2 | 10 |
| Sacrificial layers or interlayers | | | | | | |
| [30] | 98.7 | 20.8 | 60.9 | 14.5 | 1 | 10 |
| [32] | 95.0 | 37.0 | 12.6 | 17.6 | 1 | 6 |
| [33] | 97.7 | 6.5 | 40.7 | 34.0 | 1 | 6 |
| [34] | 95.5 | 11.1 | 19.9 | 42.5 | 1 | 6 |
|  | 96.8 | - | - | 35.1 | 1 | 6 |
|  | 97.2 | - | - | 33.4 | 1 | 6 |
|  | 97.3 | - | - | 31.1 | 1 | 6 |
|  | 97.3 | - | - | 29.4 | 1 | 6 |
|  | 97.4 | - | - | 28.3 | 1 | 6 |
|  | 98.1 | - | - | 26.0 | 1 | 6 |
|  | 97.8 | - | - | 20.0 | 1 | 6 |
|  | 97.2 | - | - | 32.3 | 1 | 6 |
|  | 96.7 | - | - | 37.4 | 1 | 6 |
|  | 95.3 | - | - | 43.0 | 1 | 6 |
|  | 94.6 | - | - | 45.9 | 1 | 6 |
|  | 94.3 | - | - | 43.6 | 1 | 6 |
|  | 94.2 | - | - | 40.2 | 1 | 6 |
| [59] | 96.5 | 13.4 | 24.7 | 40.3 | 1 | 6 |
| [60] | 98.9 | 39.5 | 55.0 | 7.5 | 1 | 6 |
| [61] | 99.6 | 28.6 | 178.5 | 12.1 | 1 | 3 |
| [62] | 98.9 | 21.2 | 72.3 | 15 | 1 | 2 |
| [63] | 99.2 | 41.1 | 73.6 | 26.4 | 2 | 10 |
| [64] | 98.9 | 7.9 | 84.0 | 35.7 | 1 | 4 |
| Incorporation of nanomaterials | | | | | | |
| [47] | 98.3 | 32.0 | 40.0 | 17.8 | 1 | 6 |
| [65] | 96.4 | 18.9 | 22.5 | 7.5 | 1 | 6 |
| [66] | 98.2 | 56.8 | 24 | 14.6 | 2 | 6 |
| [67] | 97.6 | 33.0 | 27.9 | 8.2 | 2 | 6 |
| [68] | 95.2 | 59.5 | 8.4 | 2.4 | 1 | 8 |
| [69] | 98.4 | 35.0 | 40.6 | 8.3 | 2 | 6 |
| [70] | 91.2 | 31.6 | 7.7 | 16.8 | 5 mM | 4.1 |
| [71] | 96.8 | 46.0 | 16.9 | 13.2 | 1 | 6 |
| [72] | 94.7 | 38.2 | 11.7 | 12.2 | 2 | 6 |
| [73] | 98.0 | 44.1 | 28.0 | 9.9 | 1 | 6 |
| [74] | 98.0 | 44.0 | 28.0 | 14.3 | - | 6 |
| [75] | 98.0 | 22.8 | 38.6 | 9.0 | 2 | 6 |
| [76] | 96.8 | 9.1 | 28.4 | 11.0 | 1 | 6 |
| [77] | 92.0 | 14.7 | 10.7 | 23.0 | 1 | 2 |
| [78] | 95.7 | 36.7 | 14.8 | 5.2 | 2 | 10 |
| [79] | 97.6 | 35.3 | 27.0 | 6.9 | 2 | 6 |
| Post-modification | | | | | | |
| [80] | 99.9 | 55.3 | 447.0 | 7.52 | 2 | 4 |
| [81] | 95.0 | 23.0 | 15.4 | 22.5 | 1 | 6 |
| [82] | 97.8 | 16.1 | 38.1 | 16.0 | 1 | 6 |
| Other methods | | | | | | |
| [7] | 99.6 | 49.6 | 126.0 | 24.8 | 2 | 4.8 |
| [15] | 99.6 | 23.2 | 217.3 | 13.8 | 1 | 6 |
|  | 99.5 | 18.3 | 181.5 | 14.9 | 1 | 6 |
|  | 99.5 | 16.5 | 153.1 | 17.5 | 1 | 6 |
|  | 99.4 | 11.4 | 148.3 | 20.9 | 1 | 6 |
|  | 97.8 | 6.0 | 43.2 | 24.5 | 1 | 6 |
| [21] | 98.0 | 42.0 | 29.0 | 10.5 | 2 | 6 |
| [29] | 96.8 | 50.0 | 15.6 | 9.8 | 1 | 6 |
| [44] | 97.6 | 12.7 | 36.4 | 13.1 | 1 | 6 |
| [48] | 96.2 | 16.0 | 22.1 | 3.3 | 1 | 6 |
| [49] | 99.6 | 22.4 | 196.0 | 20.3 | 1 | 6 |
| [83] | 90.9 | 38.2 | 6.8 | 4.4 | 1 | 7 |
| [84] | 91.1 | 35.7 | 7.2 | 13.5 | 1 | 6 |
| [85] | 99.1 | 63.6 | 40.4 | 8.5 | 1 | 5 |
| [86] | 87.7 | 30.0 | 5.7 | 1.2 | 2 | 8 |
| [87] | 88.4 | 41.5 | 5.0 | 4.2 | 1 | 6 |
| [88] | 90.0 | 16.0 | 8.4 | 10.1 | 1 | 6 |
| [89] | 99.6 | 27.0 | 182.5 | 17.1 | 1 | 4 |
| [90] | 99.1 | 27.5 | 80.6 | 25.1 | 1.5 | 4 |
| [91] | 92.1 | 37.1 | 8.0 | 26.8 | 2 | 6 |
| [92] | 98.1 | 49.0 | 26.8 | 9.7 | 1 | 4 |

^a)^PA-X nm represents the polyamide nanofilms synthesized with various thicknesses in this work.

**4. Supplementary References**

[52] Hu, J. et al. Fabrication of a high-flux sulfonated polyamide nanofiltration membrane: Experimental and dissipative particle dynamics studies. *J. Membr. Sci.* **505**, 119-129 (2016).

[53] Akbari, A. et al. Novel sulfonated polyamide thin-film composite nanofiltration membranes with improved water flux and anti-fouling properties. *Desalination* **377**, 11-22 (2016).

[54] Zhang, R. et al. A novel polyesteramide thin film composite nanofiltration membrane prepared by interfacial polymerization of serinol and trimesoyl chloride (TMC) catalyzed by 4-dimethylaminopyridine (DMAP). *J. Membr. Sci.* **542**, 68-80 (2017).

[55] Tang, Y. J. et al. Tailoring the polyester/polyamide backbone stiffness for the fabrication of high performance nanofiltration membrane. *J. Membr. Sci.* **541**, 483-491 (2017).

[56] Zhang, H. Z. et al. Highly chlorine-tolerant performance of three-channel capillary nanofiltration membrane with inner skin layer. *J. Membr. Sci.* **527**, 111-120 (2017).

[57] Wei, X. Z. et al. Negatively-charged nanofiltration membrane and its hexavalent chromium removal performance. *J. Colloid Interface Sci.* **553**, 475-483 (2019).

[58] Jiang, C. et al. Nanofiltration membranes with enhanced microporosity and inner-pore interconnectivity for water treatment: Excellent balance between permeability and selectivity. *J. Membr. Sci.* **586**, 192-201 (2019).

[59] Gao, S. et al. Ultrathin polyamide nanofiltration membrane fabricated on brush-painted single-walled carbon nanotube network support for ion sieving. *ACS Nano* **13**, 5278-5290 (2019).

[60] Wu, M. et al. Ultrathin nanofiltration membrane with polydopamine-covalent organic framework interlayer for enhanced permeability and structural stability. *J. Membr. Sci.* **576**, 131-141 (2019).

[61] Xu, S. et al. Anionic covalent organic framework as an interlayer to fabricate negatively charged polyamide composite nanofiltration membrane featuring ions sieving. *Chem. Eng. J.* **427**, 132009 (2022**).**

[62] Li, W. et al. Fabrication of high-performance nanofiltration membranes by using sulfated cellulose nanofibril as the intermediate support layer. *Desalination* **532**, 115741 (2022).

[63] Yuan, B. et al. Asymmetric polyamide nanofilms with highly ordered nanovoids for water purification. *Nat. Commun.* **11**, 6102 (2020).

[64] Han, S. et al. Covalent organic framework-mediated thin-film composite polyamide membranes toward precise ion sieving. *ACS Appl. Mater. Interfaces* **14**, 3427-3436 (2022).

[65] Lai, G. S. et al. A practical approach to synthesize polyamide thin film nanocomposite (TFN) membranes with improved separation properties for water/wastewater treatment. *J. Mater. Chem. A* **4**, 4134-4144 (2016).

[66] Wang, J. et al., Graphene oxide polypiperazine-amide nanofiltration membrane for improving flux and anti-fouling in water purification. *RSC Adv.* **6**, 82174-82185 (2016).

[67] Xie, Q. et al. Enhancing the performance of thin-film nanocomposite nanofiltration membranes using MAH-modified GO nanosheets. *RSC Adv.* **7**, 54898-54910 (2017).

[68] Lai, G. S. et al. Graphene oxide incorporated thin film nanocomposite nanofiltration membrane for enhanced salt removal performance. *Desalination* **387**, 14-24 (2016).

[69] Xue, S.-M. et al. Chlorine resistant TFN nanofiltration membrane incorporated with octadecylamine-grafted GO and fluorine-containing monomer. *J. Memb. Sci.* **545**, 185-195 (2018).

[70] Hu, R. et al. Graphene oxide-embedded polyamide nanofiltration membranes for selective ion separation. *J. Mater. Chem. A* **5**, 25632-25640 (2017).

[71] Zheng, J. et al. Sulfonated multiwall carbon nanotubes assisted thin-film nanocomposite membrane with enhanced water flux and anti-fouling property. *J. Memb. Sci.* **524**, 344-353 (2017).

[72] Sun, Z. et al. Nanovoid membranes embedded with hollow zwitterionic nanocapsules for a superior desalination performance. *Nano Lett.* **19**, 2953-2959 (2019).

[73] Ang, M. B. M. Y. et al. A facile and versatile strategy for fabricating thin-film nanocomposite membranes with polydopamine-piperazine nanoparticles generated in situ. *J. Membr. Sci.* **579**, 79-89 (2019).

[74] Xie, Q. et al. A novel double-modified strategy to enhance the performance of thin-film nanocomposite nanofiltration membranes: Incorporating functionalized graphenes into supporting and selective layers. *Chem. Eng. J.* **368**, 186-201 (2019).

[75] Bai, L. et al. Fabrication and characterization of thin-film composite (TFC) nanofiltration membranes incorporated with cellulose nanocrystals (CNCs) for enhanced desalination performance and dye removal. *Chem. Eng. J.* **358**, 1519-1528 (2019).

[76] Ji, Y. L. et al. Bio-inspired fabrication of high perm-selectivity and anti-fouling membranes based on zwitterionic polyelectrolyte nanoparticles. *J. Mater. Chem. A* **4**, 4224-4231 (2016).

[77] Wu, M. et al. Fabrication of composite nanofiltration membrane by incorporating attapulgite nanorods during interfacial polymerization for high water flux and antifouling property. *J. Membr. Sci.* **544**, 79-87 (2017).

[78] Zarrabi, H. et al. Improvement in desalination performance of thin film nanocomposite nanofiltration membrane using amine-functionalized multiwalled carbon nanotube. *Desalination* **394**, 83-90 (2016).

[79] Xue, S. M. et al. Polypiperazine-amide nanofiltration membrane modified by different functionalized multiwalled carbon nanotubes (MWCNTs). *ACS Appl. Mater. Interfaces* **8**, 19135-19144 (2016).

[80] Zhu, X. et al. Supramolecular-based regenerable coating layer of a thin-film composite nanofiltration membrane for simultaneously enhanced desalination and antifouling properties. *ACS Appl. Mater. Interfaces* **11**, 21137-21149 (2019).

[81] He, B. et al. High performance polyamide nanofiltration membranes enabled by surface modification of imidazolium ionic liquid. *J. Membr. Sci.* **608**, 118202 (2020).

[82] Peng, H. et al. Surface modified polyamide nanofiltration membranes with high permeability and stability. *J. Membr. Sci.* **592**, 117386 (2019).

[83] Qiu, W.-Z. et al. Codeposition of catechol-polyethyleneimine followed by interfacial polymerization for nanofiltration membranes with enhanced stability. *J. Appl. Polym. Sci.* **134**, 45422 (2017).

[84] Ang, M. B. M. Y. et al. Correlating PSf support physicochemical properties with the formation of piperazine-based polyamide and evaluating the resultant nanofiltration membrane performance. *Polymers* *(Basel)* **9**, 1-17 (2017).

[85] Shi, Q. et al. Poly(*p*-phenylene terephthamide) embedded in a polysulfone as the substrate for improving compaction resistance and adhesion of a thin film composite polyamide membrane. *J. Mater. Chem. A* **5**, 13610-13624 (2017).

[86] Xu, X.-L. et al. Graphene oxide nanofiltration membranes stabilized by cationic porphyrin for high salt rejection. *ACS Appl. Mater. Interfaces* **8**, 12588-12593 (2016).

[87] Qiu, W.-Z. et al. Composite nanofiltration membranes *via* the co-deposition and cross-linking of catechol/polyethylenimine. *RSC Adv.* **6**, 34096-34102 (2016).

[88] Zhang, C. et al. Polydopamine coatings with nanopores for versatile molecular separation. *ACS Appl. Mater. Interfaces* **9**, 14437-14444 (2017).

[89] Liang, Y. et al. Polyamide nanofiltration membrane with highly uniform sub-nanometre pores for sub-1 Å precision separation. *Nat. Commun.* **11**, 2015 (2020).

[90] Zhu, J. et al. Rapid water transport through controllable, ultrathin polyamide nanofilms for high-performance nanofiltration. *J. Mater. Chem. A* **6**, 15701-15709 (2018).

[91] Tang, Y. J. et al. Novel high-flux thin film composite nanofiltration membranes fabricated by the NaClO pre-oxidation of the mixed diamine monomers of PIP and BHTTM in the aqueous phase solution. *J. Membr. Sci.* **502**, 106-115 (2016).

[92] Tang, H. et al. Developing nanofiltration membrane based on microporous poly(tetrafluoroethylene) substrates by bi-stretching process. *J. Membr. Sci.* **524**, 612-622 (2017).

[93] Tanninen, J. et al. Effect of salt mixture concentration on fractionation with NF membranes. *J. Membr. Sci.* **283**, 57-64 (2006).

[94] Nicolini, J. V. et al. Selective rejection of ions and correlation with surface properties of nanofiltration membranes. *Sep. Purif. Technol.* **171**, 238-247 (2016).

[95] Li, Y. et al. Fabrication of composite polyamide/Kevlar aramid nanofiber nanofiltration membranes with high permselectivity in water desalination. *J. Membr. Sci.* **592**, 117396 (2019).

[96] Ren, T. et al. Influence of inorganic salt on retention of ibuprofen by nanofiltration. *Sep. Purif. Technol.* **189**, 382-388 (2017).
